# Neural Mechanism of Musical Pleasure Induced by Prediction Errors: An EEG Study

**DOI:** 10.3390/brainsci14111130

**Published:** 2024-11-08

**Authors:** Fuyu Ueno, Sotaro Shimada

**Affiliations:** 1 Department of Electronics and Bioinformatics, School of Science and Technology, Meiji University, Kawasaki 214-8571, Japan; fueno@meiji.ac.jp; 2 Japan Society for the Promotion of Science, Tokyo 102-0083, Japan

**Keywords:** music cognition, EEG, IDyOM, emotion, reward

## Abstract

Background/Objectives: Musical pleasure is considered to be induced by prediction errors (surprise), as suggested in neuroimaging studies. However, the role of temporal changes in musical features in reward processing remains unclear. Utilizing the Information Dynamics of Music (IDyOM) model, a statistical model that calculates musical surprise based on prediction errors in melody and harmony, we investigated whether brain activities associated with musical pleasure, particularly in the θ, β, and γ bands, are induced by prediction errors, similar to those observed during monetary rewards. Methods: We used the IDyOM model to calculate the information content (IC) of surprise for melody and harmony in 70 musical pieces across six genres; eight pieces with varying IC values were selected. Electroencephalographic data were recorded during listening to the pieces, continuously evaluating the participants’ subjective pleasure on a 1–4 scale. Time–frequency analysis of electroencephalographic data was conducted, followed by general linear model analysis to fit the power-value time course in each frequency band to the time courses of subjective pleasure and IC for melody and harmony. Results: Significant positive fits were observed in the β and γ bands in the frontal region with both subjective pleasure and IC for melody and harmony. No significant fit was observed in the θ band. Both subjective pleasure and IC are associated with increased β and γ band power in the frontal regions. Conclusions: β and γ oscillatory activities in the frontal regions are strongly associated with musical rewards induced by prediction errors, similar to brain activity observed during monetary rewards.

## 1. Introduction

Humans can experience pleasure from a wide range of stimuli and activities, from primary rewards that satisfy basic biological needs, such as food, to aesthetic experiences like music appreciation [1]. Among these, music is one of the most potent sources of pleasure for many individuals [2], with a unique process through which pleasure is evoked [3]. This is considered to be due to the highly dynamic temporal structure of music, where acoustic features change across timescales from milliseconds to several minutes, causing rapid shifts in emotional responses [4,5]. Nevertheless, few studies have comprehensively examined the pleasure induced by music and the underlying neural mechanisms, specifically in relation to how changes in the temporal structure of music elicit pleasure experiences.

Studies in which a combination of positron emission tomography and functional magnetic resonance imaging (fMRI) was used have shown that dopamine is released in the striatum during anticipation and peak experience of musical pleasure, which induces chills [6]. Here, “chills” represent an embodied peak emotional experience characterized by goosebumps and dopamine release [6,7]. Chills are widely regarded as a marker of the highest pleasure levels [8] and can be induced not only by music but also by other stimuli, such as films and speeches [7]. Given that chills are positively correlated with both valence and arousal [7], we define “musical pleasure” in this study as a state of high valence and high arousal induced by music, which at times reaches the highest level of pleasure (i.e., chills). It should be noted that positive emotions, such as enjoyment of a sunny walk or the satisfaction of successfully controlling a robotic hand [9], are often associated with high valence alone [10], and, in cases of low arousal, such experiences are not considered “pleasure” within the scope of this study.

It has been suggested that musical pleasure is caused by predictive mechanisms [3,11,12]. Notably, listeners possess internal predictive models, which involve learning from their diverse experiences and habits across various music genres [3]. They gather cues from the music they listen to in real time and use their internal predictive models to anticipate how upcoming acoustic features will change [13,14,15]. This prediction inherently involves uncertainty. When surprise is due to a deviation from the prediction (prediction error), the brain’s reward system is activated, eliciting pleasure [16,17].

Cheung et al. [16] employed a statistical learning model, the Information Dynamics of Music (IDyOM) [13], to calculate the uncertainty and surprise associated with 80,000 chords from U.S. Billboard pop songs. Their findings revealed that high subjective pleasure is induced when chords greatly deviate from the listeners’ expectations or conform to expectations in cases of informational ambiguity. Furthermore, fMRI data have indicated the involvement of the amygdala, hippocampus, auditory cortex, and nucleus accumbens in this process [16]. Shany et al. [17] investigated neurobehavioral responses to surprise across three musical pieces (classical and film music) using fMRI and subjective ratings of emotional valence and arousal. Their results showed that musical surprise, compared to unsurprising events, led to increased activation in the auditory cortex, insular cortex, and ventral striatum (VS), which was associated with changes in emotional valence and arousal. Furthermore, compared with the participants who experienced less musical pleasure, those who experienced greater musical pleasure exhibited stronger surprise-related connectivity between the nucleus accumbens and auditory cortex during the most pleasant piece [17]. Therefore, musical surprise plays a crucial role in producing musical pleasure.

Musical surprise can be continuously estimated using the IDyOM model [13,18], which is a variable-order Markov model of expectation. Using this approach, Omigie et al. [19] found that as the estimated surprise of notes increased, the amplitude of the N1 component of the ERP also increased, suggesting that it is possible to record the neural responses to subtle changes in auditory expectations [20]. The IDyOM model has proven highly reliable in predicting the listeners’ explicit ratings of surprise and uncertainty [21,22,23] and has also demonstrated high discriminative power, successfully simulating other music cognition processes [24], including predicting the listeners’ style-specific expectations [21], recognition memory [25], perceptual similarity [26], and metrical inference [27].

However, simple auditory stimuli have been used in previous studies in which the IDyOM model was used, focusing exclusively on melody [18,22,23,28,29] or chord progression [16]. Furthermore, specific genres, such as Western folk songs and hymns, were used in many of these studies [18,22,23,28,29]. The tendency to use only specific genres of music has also been observed in previous studies on musical rewards. Notably, the prevalence of studies in which only classical music was used is overwhelming [8,30,31,32,33,34,35,36,37]. In some studies, film and classical music [17], pop/rock [38], and only pop [16] have been incorporated. Notably, various genres of music have been used in some studies [6,39]; however, only a specific genre has been used in most studies. It is challenging to generalize the findings of studies in which only a specific genre of music was used because musical structures and other elements vary across genres. To address these issues, incorporating music from various genres rather than simple melodies or chords from a specific genre is necessary. Furthermore, extracting melodies and harmonies as closely as possible from their original forms is essential. Further investigation into the relationship between the musical surprise derived from these elements and pleasure is warranted. In the study of Salimpoor et al. [6], various musical genres were used. However, the pieces used in the study were provided by the participants, who chose tracks that induced strong emotions of pleasure and chills. This suggests that familiarity with music may influence the induction of pleasure [3]. Therefore, to eliminate the potential influence of varying degrees of familiarity among the participants, using music that the participants have never heard is necessary. This approach allows the investigation of pleasure induced purely by surprise owing to prediction error.

Furthermore, as previously mentioned, it has been suggested in fMRI and other neuroimaging studies that prediction errors induce musical pleasure. However, how temporal changes in musical features are involved in musical reward processing remains unclear. To address this issue, an EEG-based neuroimaging approach has been considered effective. This is because EEG provides millisecond-level fine temporal cerebral information, which other neuroimaging methods, such as fMRI, cannot offer [40]. Considering the rapid temporal changes in musical features, EEG is a suitable approach. In EEG-based studies, components such as mismatch negativity and early right anterior negativity reflect the prediction errors induced by music [41,42,43,44,45,46]. However, whether these prediction errors induce pleasure cannot be determined using ERPs alone. Therefore, in the present study, we employed approaches involving analysis of oscillatory activities in response to monetary rewards in gambling tasks [47,48] and vicarious rewards in competitive games [49]

Marco-Pallares et al. [47] conducted an experiment using a gambling task in which the value (reward/monetary gains or punishment/monetary losses) was always aligned with correctness (correct or incorrect choice). They reported that after receiving feedback on the outcome of the trials, an increase in β power at Fz was observed in gain trials than in loss trials [47]. Mas-Herrero et al. [48] conducted an EEG and fMRI experiment using the same gambling task and integrated time–frequency oscillation EEG data with fMRI activity using Joint Independent Component Analysis (ICA). The findings revealed that the beta oscillatory activity (BOA) in the frontal region increased after the participants received positive feedback, which was associated with the ventral striatum, bilateral hippocampus, and prefrontal cortex [48]. Inomata et al. [49] investigated brain activity using EEG while the participants observed their preferred player winning a competitive (rock–paper–scissors) game. The results indicated that the BOA in the frontal region was activated when a participant’s preferred player won (vicarious reward). Furthermore, current source localization using standardized low-resolution electromagnetic tomography (sLORETA) revealed that the BOA originated from the posterior part of the anterior cingulate cortex (ACC) [49]. fMRI-based studies have demonstrated that the VS, ventromedial prefrontal cortex, and ACC are involved in reward processing [50,51,52,53,54]. In Marco-Pallares et al.’s study, after receiving feedback on the outcome of the trials, an increase in θ power at the frontal central electrodes was observed in loss trials compared with gain trials [47]. Furthermore, β-γ oscillatory activity has been reported to increase after unexpected monetary rewards [55].

Therefore, in the present study, we aimed to quantitatively calculate musical surprise induced by prediction errors using the IDyOM model based on the melodic and harmonic features of music. To achieve this goal, we used music from various genres the participants had never heard. These pieces comprised the stimuli in the EEG experiment conducted while the participants listened to music. Then, we used time–frequency analysis of EEG data to investigate whether brain activity during musical pleasure induced by predictive mechanisms would be similar to brain activity observed when monetary rewards are received in gambling tasks. Specifically, we hypothesized that similar to the findings from studies using gambling tasks mentioned earlier, music listening would evoke pleasure through prediction errors (musical surprise), resulting in a decrease in frontal θ power and an increase in β and γ power. In addition, we aimed to examine whether musical surprise derived from melody or harmony would be strongly associated with pleasure across various music genres. We speculated that conducting these investigations would contribute to elucidating the mechanisms of reward processing in music.

## 2. Materials and Methods

### 2.1. Participants

Thirty-two healthy adults were initially recruited for this study. After pre-screening, twenty-four participants with a publicly available Barcelona Music Reward Questionnaire (BMRQ) [56] score of >65, indicating a heightened sensitivity to music rewards [57,58], took part in the experiment. All participants were Japanese nationals, and their native language was Japanese. Additionally, we confirmed that all the participants met the criteria, which included not being returnees and not having confidence in their English listening skills. This was important because English songs were used as musical stimuli in this study. Two participants were excluded because of technical issues caused by equipment malfunction during the EEG data recording. Four participants were excluded because of excessive noise. One participant who fell asleep during the experiment and two who understood the lyrics of certain pieces of music were also excluded. Therefore, 15 participants were finally included in the analysis (four female; age [mean ± standard deviation (SD)]: 23.3 ± 3.41 years). There were no professional musicians among the participants; however, six had received formal training in musical instruments, including voice (1 year [*n*= 1], 3–5 years [*n* = 4], >10 years [*n* = 1]). All participants provided written informed consent to participate in this study. The study protocol was approved by the Ethics Committee of the School of Science and Technology, Meiji University (Approval Code: 24-575, Approval Date: 25 May 2024). This study was conducted according to the principles and guidelines of the Declaration of Helsinki.

### 2.2. EEG Recordings

The Brain–Computer Interface Research System, an EEG measurement system manufactured by g.tec (Schiedlberg, Austria), was used for the EEG measurements. A bioamplifier for EEG measurements (g.USBamp; g.tec, Schiedlberg, Austria) was used, and sintered silver [Ag]/Ag chloride [AgCl] electrodes from the same measurement system were used. The electrodes used were an active scalp electrode (g.LADYbird; g.tec, Schiedlberg, Austria) and a reference electrode (g.GAMMAearclip Ag/AgCl; g.tec, Schiedlberg, Austria). The scalp and ground electrodes were flat, whereas the reference electrode was an ear-clip type. A gamma box for direct current (g.GAMMAbox for 16 channels DC; g.tec, Schiedlberg, Austria) was used to connect the bioamplifier and electrodes. In addition, electro-oculography (EOG) was performed simultaneously with EEG measurements from the same bioamplifier. A 0.5–100 Hz bandpass filter was applied to the EEG and EOG, and the sampling frequency was recorded at 512 Hz.

Two personal computers (PCs) were used in the experiment—a measurement PC to measure the EEG data and a control PC to run the experimental program. The bioamplifiers were controlled and measured using numerical analysis software (MATLAB R2018b; MathWorks, Natick, MA, USA) and Simulink on the measurement PC. Experimental programs were created and executed on the control PC using the psychological experiment software E-prime 3.0 (Psychology Software Tools, Sharpsburg, PA, USA) and Presentation 24.0 (Neurobehavioral Systems, San Francisco, CA, USA). These software were used to present the musical stimuli. E-prime 3.0 (Psychology Software Tools) was utilized during the EEG measurement sessions while the participants listened to music. In this session, to record the timing information while the participants listened to the music, trigger signals with changing values at the start and end of each piece were output via the parallel port of the control PC. These signals were input into the bioamplifiers through a trigger cable. The EEG data, synchronized with the timing information, along with the trigger data were then recorded on the measurement PC via a USB connection from the bioamplifers. Presentation 24.0 (Neurobehavioral Systems) was utilized during the sessions of the real-time reports of pleasure values while the participants listened to music. In this session, only the control PC was used to record the pleasure values entered by the participants during music listening, along with the corresponding timing information of the music.

In the EEG measurements, electrodes were mounted at 30 positions according to the international 10–20 system (Fp1, Fp2, F7, F3, Fz, F4, F8, FT7, FC3, FCz, FC4, FT8, T7, C3, Cz, C4, T8, TP7, CP3, CPz, CP4, TP8, P7, P3, Pz, P4, P8, POz, O1, and O2). First, Cz was determined from the midpoint of the line connecting the nasal root and occipital tubercle and the midpoint connecting the left and right anterior auricular points. An electrode cap was then placed over the entire head, with Cz as the reference. The ground electrode was located at AFz, the reference electrode was mounted on the left earlobe, and the montage was recorded using the reference electrode derivation method. Vertical EOGs were recorded from above and below the left eye.

### 2.3. Analysis of Musical Surprise

We used version 1.7.1 of the IDyOM model [13,18] to obtain an objective measure of musical surprise. The IDyOM model is a variable-order Markov model that quantifies continuous surprise as information content (*IC*). Using the IDyOM model, we calculated the *IC* of musical pieces. Musical surprise, which differs from the “subjective surprise” that listeners may experience, was objectively measured based on the musical features of the piece. The IDyOM model incorporates a short-term (stm) model that generates dynamic expectations based on recent auditory sequences and a long-term (ltm) model that generates canonical expectations derived from extensive training data [20]. We employed a configuration combining both the ltm and stm, known as “both+”. This involved training the model using extensive training data and recent auditory sequences while incorporating the latter into the ltm model [59]. The *IC* is calculated using the following equation:

$$IC_{p}=-\log_{2}p$$

where *p* indicates the conditional probability of the current auditory event, considering the preceding auditory events and the model’s long-term learning. This equation indicates that as the probability of an event decreases, its *IC* value increases. This relationship leads to listener surprise.

We utilized the Real World Computing research music database (https://staff.aist.go.jp/m.goto/RWC-MDB/index-j.html (accessed on 6 November 2024)) to calculate the *IC*. Notably, most musical pieces in this database are original and not widely distributed, indicating that all the participants were encountering these musical pieces for the first time, which is why we chose them for this study. We adopted a set of 100 popular musical pieces from this database as training data. This decision was based on the belief that most people are familiar with popular music. Therefore, listeners can predict the next auditory event based on their listening experience. Furthermore, we adopted 70 musical pieces across six genres (Pop, Rock, Dance, Jazz, Latin, and World) as target data from the database. The remaining 30 musical pieces, including classical and Japanese music, were not selected. Classical pieces are existing compositions that are potentially familiar to the participants. Japanese music was not chosen because of the potential confounding effect of lyrics. The 70 selected pieces included instrumental pieces and songs with lyrics, which were in English. The six genres for the target data were selected to analyze the relationship between pleasure and surprise across diverse musical characteristics. This analysis aimed to reveal common phenomena independent of genre-specific features.

Herein, we describe the detailed procedure of our analysis. From among the two types of music data, the Musical Instrument Digital Interface (MIDI) format and compact disc (CD) audio, we used the MIDI data for this analysis. First, for each musical piece, we used MuseScore 3.6.2 (https://musescore.org/en (accessed on 6 November 2024)), software capable of editing music. Using this software, we created separate data for the melody and harmony, allowing us to calculate the *IC* for both melody and harmony. We used these data for subsequent analysis. However, raw data are often unsuitable for direct analysis, particularly for harmony. Therefore, we edited the data, ensuring that notes smaller than the 16th note, tuplets with more than three notes, and decorative notes, such as glissandos, were eliminated. The editing process also involved partially deleting notes, altering note lengths, or shifting beats as necessary while striving to preserve the original form of the piece as much as possible. In addition, for the harmony data, we reduced the number of parts (such as violin, guitar, and bass) to four or five whenever feasible. We also edited the data such that three to five notes constituted a single chord, sounding as simultaneously as possible.

Second, musical data edited into an analyzable form were used to analyze the *IC* of melody and harmony. In this process, as mentioned previously, the training data consisted of popular music, whereas the target data consisted of music across six genres. However, some pieces could not be analyzed and were excluded. Notably, pieces with numerous tuples or off-beat sections in improvisations could not be adequately edited without distorting the original composition. Finally, the *IC* values were output with temporal information, which was subsequently modified. Because the experimental stimuli used CD audio sources, the temporal information calculated from the MIDI data tempo was adjusted to match the temporal information from the CD audio sources. The reason for using CD audio sources rather than MIDI data as experimental stimuli was to analyze the acoustic features influenced by rich and non-monotonous expressions of human performance in future studies. This analysis aimed to relate these features to the findings of the present study and uncover new findings.

### 2.4. Music Stimuli

Based on the *IC* analysis results, pieces used as experimental stimuli were selected based on two criteria. The primary criterion was the range of *IC* values, ensuring that they ranged from low to high to elucidate the relationship between surprise and pleasure. The secondary criterion was the number of editing points in the music, ensuring they were minimal for utilizing the most accurate *IC* values possible. Based on these criteria, eight musical pieces were selected as experimental stimuli, ensuring at least one musical piece from each genre. The total length of all musical pieces was approximately 30 min. Table 1 presents the details of the eight selected musical pieces. The standard deviations of the *IC* for melody for the eight musical pieces were as follows: Clip1: 3.37, Clip2: 5.24, Clip3: 3.07, Clip4: 5.18, Clip5: 5.37, Clip6: 5.38, Clip7: 4.14, and Clip8: 4.16. The standard deviations of the IC for harmony for the eight musical pieces were as follows: Clip1: 1.54, Clip2: 1.43, Clip3: 1.94, Clip4: 1.84, Clip5: 1.9, Clip6: 1.45, Clip7: 1.46, and Clip8: 1.4.

The CD audio sources of the eight pieces were imported as Waveform Audio File Format into audio editing software (Audacity 3.3.3; https://www.audacityteam.org/ (accessed on 6 November 2024)). The loudness of all music was normalized to ensure the maximum amplitude reached −1 dB. Furthermore, the first 1 s of the music was processed with a fade-in, whereas the last 1 s was processed with a fade-out.

### 2.5. Procedure

We used E-prime 3.0 and Presentation 24.0 to control the experimental procedure. In addition, a 27-inch liquid crystal display monitor and keyboard were used to present the fixation cross and collect inputs for subjective pleasure. Before conducting the experiment, we obtained the three-dimensional (3D) coordinates of the electrodes for each participant. The participants wore an M-sized electrode cap (g.tec), and measurements were obtained using a 3D digitizer (FASTRAK, Polhemus, VT, USA). During the experiment, the participants were seated in front of the monitor, and their EEG data were recorded while listening to the presented music. The participants listened to the presented music using earphones (SE846, SHURE, Niles, IL, USA).

**Figure 1:**
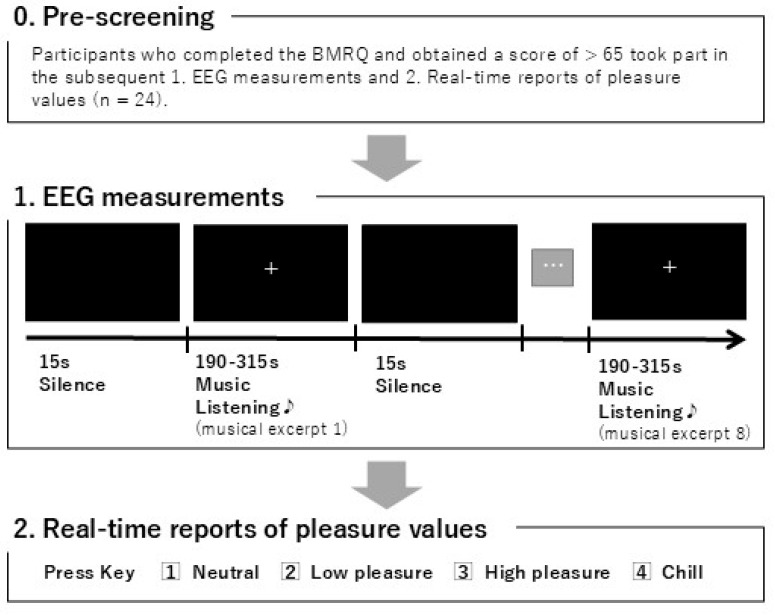
Procedure from the participants’ pre-screening to the end of the experiment.

First, EEG data were recorded while the participants listened to the eight musical pieces. While listening to the musical piece, the participants were instructed to gaze at a fixation cross. A cycle consisting of a 15 s silence period followed by the presentation of musical stimuli for the duration of each piece was repeated eight times (for the eight musical pieces). The eight musical pieces were randomly presented to the participants. After the participants had listened to all eight pieces, and the EEG measurements had been obtained, a 5-min break was observed. Next, real-time reports of pleasure values were collected while the participants listened to the eight musical pieces again. The cycle was essentially the same as that described earlier; however, during the presentation of the musical stimuli, the participants reported the degree of pleasure experienced in real-time on a scale of 1–4 (1 = neutral, 2 = low pleasure, 3 = high pleasure, 4 = chills; [8]) using a keyboard. “Neutral” was reported when no pleasure was felt, “pleasure” and “chills” were reported when valence as well as arousal were high [7], and “chills” was reported in particular when there was a sensation of goosebumps [6]. The participants were instructed to continuously press one of the keys from 1 to 4 at all times. They were required to press 1 when they first heard the music and release their finger from the key when it ended and was no longer audible. There were no specific instructions regarding the finger or hand (right or left) that the participants should use to press the keys. During both the EEG measurement sessions and the real-time reports of pleasure values sessions while listening to music, the participants were given the following two instructions. First, they were instructed to minimize body movement as much as possible (for instance, avoiding foot-tapping in time with the rhythm). This was to prevent noise in the EEG data and to avoid any influence on the degree of pleasure caused by moving in time with the music. Second, the participants were instructed to not try to infer the meaning of the English lyrics but rather to listen to the music as a whole. This was to ensure that pleasure was not influenced by the meaning of the lyrics. Figure 1 shows the procedure from the participants’ pre-screening to the end of the experiment.

### 2.6. Data Analysis

The EEG data were preprocessed using MATLAB (MathWorks) and EEGLAB 14.1.1b (Swartz Center for Computational Neuroscience, San Diego, CA, USA) [60]. First, we extracted the EEG data corresponding to the entire music listening period from the raw data. This extraction was based on the trigger signals recorded at the start of the first music listening session and the end of the eighth session. Second, the data were subjected to 1–60-Hz bandpass and 50-Hz notch filters. Third, the electrode information collected for each participant, as described in Section 2.5, was recorded. Notably, the electrode positions measured using a digitizer (Polhemus) were registered using sLORETA [61] to fit the coordinates of a standard brain to the Montreal Neurological Institute coordinate system. Fourth, the participants with data containing excessive noise segments were excluded (see Section 2.1. Participants for details). The reason for this exclusion is that, in this study, each piece of music was measured only once per participant, and all data needed to be maintained under the same conditions. Therefore, when excessive noise was observed, channel or time segment rejection was not performed; instead, data from these participants were removed entirely. Specifically, participants were excluded if their data included segments that met both of the following criteria when compared to the overall voltage trends: (1) steep and excessive changes, and (2) abnormal spatial distribution (either limited to a single electrode or excessively widespread). Fifth, re-reference was performed using the average reference method across all electrodes. Finally, we conducted Infomax ICA, specifically using ‘runica’ with default options. We re-referenced to the average after including the initial reference, ensuring a full rank of the data [62]. Among the first generated components, in most cases, there were typically two artifact components corresponding to blinks and eye movements, characterized by a clear distribution over the frontal region and a smoothly decreasing EEG spectrum. These artifact components were rejected. We also rejected muscle artifact components, characterized by spatial localization and high power at high frequencies (above 20–30 Hz). The EEG data after rejection were used for the subsequent analysis.

Next, a time–frequency analysis was performed on the preprocessed EEG data. The analysis was performed on the data during music listening (for the duration of each piece) using complex Morlet wavelets $w\;\left(t,\;f_{0}\right)$. It was centered around the central frequency $f_{0}$ with a Gaussian shape, having SD $\sigma_{t}$ and SD $\sigma_{f}$ in the time and frequency domains, respectively, as described in the following equation [63].

$$w\;\left(t,\;f_{0}\right)={\left(\sigma_{t}\sqrt{\pi }\right)}^{-\frac{1}{2}}\exp\left(-\frac{t^{2}}{2{\sigma_{t}}^{2}}\right)\exp\left(2i\pi f_{0}t\right)$$

The wavelet family, characterized by the constant ratio $\frac{f_{0}}{\sigma_{f}}\;\left(\sigma_{f}=\frac{1}{2\pi \sigma_{t}}\right)$, was set to 7. The time-varying energy $E\left(t,\;f_{0}\right)$ was calculated using signal $s\left(t\right)$ from 1 to 45 Hz in 1 Hz steps using the following equation:

$$E\left(t,\;f_{0}\right)={\left|w\left(t,f_{0}\right)\times s\left(t\right)\right|}^{2}$$

Then, the power values for each frequency were calculated and averaged across each frequency band (δ band: 1–3 Hz, θ band: 4–7 Hz, α band: 8–13 Hz, β band: 14–30 Hz, and γ band: 31–45 Hz) to obtain the power value for each band.

In particular, we focused on the power values for the θ, β, and γ frequency bands (particularly the β band) in the frontal region (Fp1, Fp2, F3, Fz, F4, FC3, FCz, and FC4). These bands in the frontal region are suggested to be associated with activity in the reward system of the brain [47,48,49,55]. Therefore, for each frequency band (θ, β, and γ), we calculated the average power values across the eight frontal electrodes mentioned earlier.

To analyze the associations of brain activity with pleasure and surprise, we used a general linear model (GLM), which comprises an analysis method that statistically examines how well the observed signal data can be fitted to a design matrix model [64]. We fitted the time-course data of subjective pleasure or *IC* (for melody/harmony) as the design matrix model to the time course of the aforementioned average power values. Both the time-course data for subjective pleasure or *IC* and the time course of the power values were derived by averaging the original data at 1 s intervals. Then, t-values indicating how well the EEG power values fit subjective pleasure or *IC* were calculated. For the detailed equations, please refer to the study of Ueno and Shimada [65].

Owing to variations in music length and data size across different musical pieces, the t-values were converted to z-scores for each piece. Finally, for each frequency band, a t-test against zero was performed on the z-scores across all musical pieces and participants (eight pieces × 15 participants). This analysis allowed us to examine whether there was a significant relationship between frontal power values in the θ, β, and γ frequency bands and subjective pleasure or *IC* (for melody/harmony), based on data from all musical pieces.

## 3. Results

**Figure 2:**
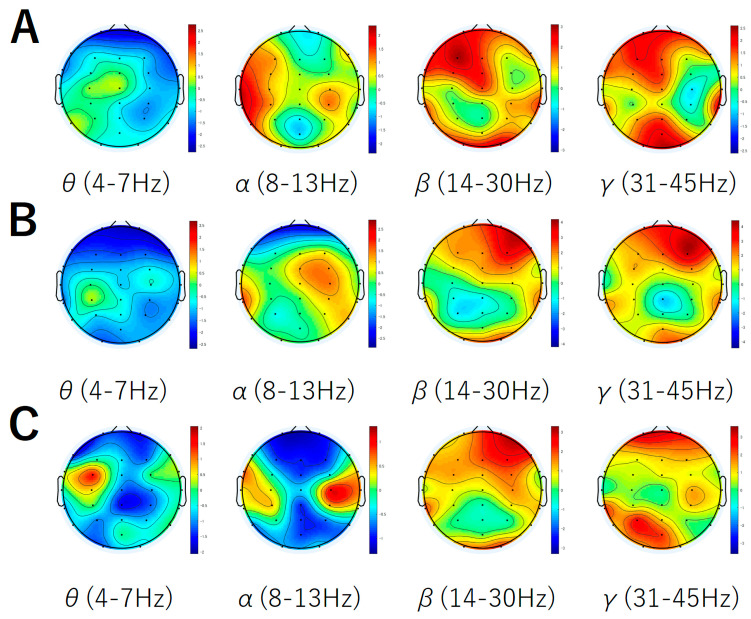
Topographic map showing the GLM analysis results with power values. These topographic maps were created based on the t-values derived from the GLM analysis. These t-values were calculated from the GLM analysis performed on the data for all combinations of musical pieces and participants (eight pieces × 15 participants). (**A**) Results on the time course of the power values and the time-course data of subjective pleasure in the θ, α, β, and γ bands. (**B**) Results on the time course of the power values and the time-course data of the *IC* for melody in the θ, α, β, and γ bands. (**C**) Results on the time course of the power values and the time-course data of the *IC* for harmony in the θ, α, β, and γ bands.

The results are shown in Figure 2A–C. Figure 2A shows a topographic map of the results from a GLM analysis of the time course of the power values and the time-course data of subjective pleasure (the results for the α band are also shown). Figure 2B shows a topographic map of the results from a GLM analysis of the time course of the power values and the time-course data of the *IC* for melody (the results for the α band are also shown). Figure 2C shows a topographic map of the results from a GLM analysis of the time course of the power values and the time-course data of the *IC* for harmony (the results for the α band are also shown). These topographic maps were created based on the *t*-values derived from the GLM analysis. These t-values were calculated from the GLM analysis performed on the data for all combinations of musical pieces and participants (eight pieces × 15 participants). For further details, please refer to Section 2.6 Data Analysis. Supported by these topographic maps and previous research findings [47,48,49,55], we focused our investigation on the activity of the frontal region.

A GLM analysis of the time course of the average power values across the frontal region (Fp1, Fp2, F3, Fz, F4, FC3, FCz, and FC4) and the time-course data of subjective pleasure revealed the following findings. A significant positive fit was observed in the β (*t*(212) = 4.12, *p* < 0.01) and γ (*t*(212) = 3.32, *p* < 0.01) bands. However, no significant fit was observed in the θ band (*t*(212) = −0.457, *p* > 0.1). There were 13 musical piece–participant combinations in which the time course of subjective pleasure during listening to music was consistently reported as 1 (neutral) from beginning to end. For these datasets, the GLM fit results could not be obtained, resulting in the previously mentioned t-values lacking degrees of freedom. However, the amount of data for which results could not be obtained did not differ significantly among the eight musical pieces.

A GLM analysis of the time course of the average power values across the frontal region (Fp1, Fp2, F3, Fz, F4, FC3, FCz, and FC4) and the time-course data of the *IC* for melody revealed the following findings. A significant positive fit was observed in the β (*t*(238) = 4.58, *p* < 0.01) and γ (*t*(238) = 4.12, *p* < 0.01) bands. However, no significant fit was observed in the θ band (*t*(238) = −0.978, *p* > 0.1).

A GLM analysis of the time course of the average power values across the frontal region (Fp1, Fp2, F3, Fz, F4, FC3, FCz, and FC4) and the time-course data of the *IC* for harmony revealed the following findings. A significant positive fit was observed in the β (*t*(238) = 2.99, *p* < 0.01) and γ (*t*(238) = 3.19, *p* < 0.01) bands. However, no significant fit was observed in the θ band (*t*(238) = −0.787, *p* > 0.1).

Subsequently, we investigated the correlation between subjective pleasure and *IC* (for melody/harmony). To enhance understanding of their temporal progression and relationship, we presented the following figures.

**Figure 3:**
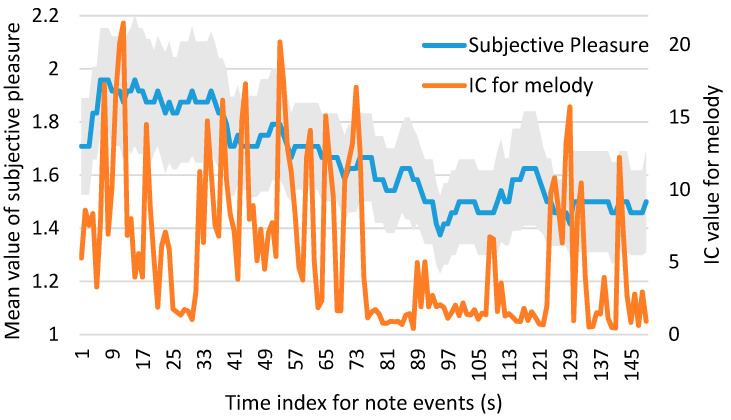
The time-series plot of the average subjective pleasure across all participants and *IC* for melody in Clip 4. The left vertical axis represents the values of subjective pleasure, while the right vertical axis represents the values of *IC* for melody. The horizontal axis represents the time index for note events. Since *IC* values are calculated for each note, the plots only show *IC* values and corresponding subjective pleasure values for periods during which a melody is present. The standard error range is indicated by gray shading.

**Figure 4:**
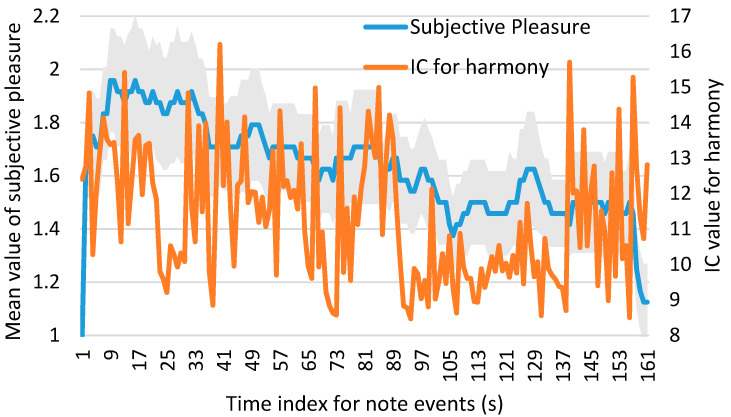
The time-series plot of the average subjective pleasure across all participants and *IC* for harmony in Clip 4. The left vertical axis represents the values of subjective pleasure, while the right vertical axis represents the values of *IC* for harmony. The horizontal axis represents the time index for note events. Since *IC* values are calculated for each note, the plots only show *IC* values and corresponding subjective pleasure values for periods during which a harmony is present. The standard error range is indicated by gray shading.

Figure 3 and Figure 4 show the time-series plots of subjective pleasure and *IC* (for melody/harmony) for Clip 4 as examples. The left vertical axis represents the values of subjective pleasure, while the right vertical axis represents the values of *IC* (for melody/harmony). The horizontal axis represents the time index for note events. Since *IC* values are calculated for each note, the plots only show *IC* values and corresponding subjective pleasure values for periods during which a melody or harmony is present. Therefore, the maximum time index does not reflect the total length of the piece, and the time index is different between *IC* for melody and *IC* for harmony even for the same piece. The standard error range is indicated by gray shading. Figure 3 shows the time-series plot of the average subjective pleasure across all the participants and *IC* for melody. Figure 4 shows the time-series plot of the average subjective pleasure across all the participants and *IC* for harmony.

Spearman’s rank correlation coefficient was calculated between subjective pleasure and *IC* (for melody/harmony) using data from all musical pieces. There was a weak correlation between subjective pleasure and the *IC* for melody (*r* = 0.0961, *p* < 0.01). The correlation between subjective pleasure and the *IC* for harmony was not statistically significant (*r* = 0.0286; *p* > 0.1).

## 4. Discussion

In the present study, we investigated whether brain activity experienced during musical pleasure induced by predictive mechanisms was similar to that experienced during other rewarding experiences, including receiving monetary rewards in gambling tasks, using a time–frequency analysis of EEG data. We found that both subjective pleasure and *IC* were associated with an increase in β and γ band power in the frontal regions. These activities are consistent with brain activities observed during the receipt of monetary rewards in gambling tasks [47,48,55] and vicarious rewards in competitive games [49]. However, we found that neither subjective pleasure nor *IC* was associated with the θ band power in the frontal regions. This result alone was not consistent with the brain activity observed during the receipt of monetary rewards, as previously mentioned.

Based on the findings of the previous studies, it can be inferred that an increase in β power in the frontal region is involved in the reward experience associated with positive outcomes in tasks such as gambling. Furthermore, it has been reported that β-γ oscillatory activity increases after obtaining rewards in gambling and learning tasks [47,66,67,68]. It has also been reported that β-γ oscillatory activity is not activated by outcomes worse than expected but by outcomes better than expected [55,69]. β-γ oscillatory activity has been reported to occur in the ventromedial prefrontal cortex [55]. HajiHosseini et al. [69] investigated prediction errors using a gambling task in which cues indicated the probability and magnitude of upcoming outcomes (monetary gains or losses). They found that increases in β-γ oscillatory activity were observed only after low-probability gains [69]. The increase in β-γ oscillatory activity after unexpected rewards has also been reported in previous studies [70,71]. γ oscillatory activity has been associated with attention [72] and novelty detection [73]. Therefore, the concurrent increase in γ oscillatory activity with BOA in the frontal cortex may be associated with reward processing, enhancing attention to unexpected stimuli, triggering reward system activity, and increasing emotional responses to such stimuli [55].

Furthermore, Marco-Pallares et al. [47] investigated θ oscillatory activity and reported that after receiving feedback on the outcome of the trials in a gambling task, an increase in θ power at the frontal central electrodes was observed in loss trials than in gain trials [47]. Such frontal θ activity increases more with negative feedback than with positive feedback [48,55,70,74,75,76]. In addition, θ oscillatory activity associated with negative feedback is considered to originate from the dorsal anterior cingulate cortex [55]. Error-related negativity (ERN) in response to performance errors has been indicated in studies in which ERP was used, and medial frontal negativity (MFN) has been observed after feedback indicating an incorrect response [77] or monetary loss in gambling tasks [78]. Furthermore, a significant increase in θ band power is observed in loss (negative) feedback compared with gain (positive) feedback, suggesting that the θ band is involved in the generation of ERN and MFN [47,78,79]. Therefore, positive outcomes are considered to result in a decrease in frontal θ activity compared with negative outcomes. Thus, we hypothesize that a similar decrease may occur not only with monetary rewards but also with musical rewards.

However, different findings regarding θ oscillatory activity have been observed in studies on music and emotional responses. An increase in θ activity has been associated with music with positive valence [80,81,82] or consonant music perceived as pleasant [83]. Furthermore, an increase in θ phase-synchronization between electrodes over the right temporal and frontal regions has been associated with heightened musical pleasure [39]. These findings are considered relevant to understanding differences in the θ activity associated with positive outcomes in monetary rewards discussed earlier in relation to previous studies.

Ali Diez et al. [2] modified the Monetary Incentive Delay task [84] for EEG and conducted an experiment using a cue–target–feedback structure with two types of stimuli—monetary and music. They aimed to investigate the neural mechanisms underlying reward anticipation and consumption. Notably, the participants were presented with cues regarding potential future outcomes. Feedback, in the form of “monetary gain/pleasant music listening or monetary loss/unpleasant music listening”, was provided based on quick responses to subsequent target stimuli. In both monetary and music conditions, θ activity increased more during the anticipation of a reward (cue gain) compared to the anticipation of a loss (cue loss). Conversely, at the time of feedback, for monetary outcomes, θ activity decreased during reward gain (positive feedback) than during reward loss (negative feedback) under both the cue gain and loss conditions. However, for music outcomes, θ activity increased during negative feedback in the cue gain condition, while it increased during positive feedback in the cue loss condition. Therefore, it was suggested that while θ activity showed similar patterns between monetary and music stimuli during the anticipation phase, different outcomes were observed during the reward consumption phase [2].

Based on the findings of these previous studies and our results, it is suggested that the increase in frontal β and γ power is associated with pleasure and surprise (prediction error) and that the results for music and monetary rewards are consistent. However, θ power does not appear to show the same consistency between music and monetary rewards, suggesting that θ power may not be involved in music rewards. Since there have been relatively few studies using time–frequency analysis of EEG in relation to musical rewards, further research will be needed to clarify how, or whether, θ power is related to musical rewards or surprise.

Previous studies have reported that mismatches in anticipated events enhance attentional processing, increase physiological arousal, and induce dopaminergic activity in the ventral tegmental area of the midbrain reward pathway, thereby amplifying emotional responses to these events [55]. Additionally, β-γ oscillatory activity has been suggested to play an important role in the brain mechanisms underlying attention, reward, and memory, particularly through interactions between the frontal cortex and striatum, as well as between the hippocampus and striatum [48,55]. Thus, as individuals listen to music in real time, when they predict how upcoming acoustic features will change and encounter prediction errors (surprises), it is probable that the neural mechanisms involved in attention, reward, and memory are engaged, reflected in β-γ oscillatory activity when musical pleasure is experienced.

Furthermore, we investigated whether musical surprise (*IC*) derived from either melody or harmony in various music genres is closely associated with pleasure. We found that brain activity associated with subjective pleasure was similar to that associated with *IC*. Therefore, it can be implied that musical surprise (*IC*) derived from either melody or harmony in various music genres is closely associated with pleasure. However, a weak or non-significant correlation was found between subjective pleasure and *IC* (for melody/harmony). The reason for this is that, while the *IC* is calculated directly from the music data, there is no time delay. However, subjective evaluations require the participants to press a key to evaluate the degree of pleasure they experience while listening to the music. Since they respond after perceiving changes in their emotion, a time lag of a few seconds may occur in their responses. This difference in timing between subjective pleasure and *IC* can also be observed in Figure 3 and Figure 4, suggesting that there may be a temporal offset between the two measures. Future studies might observe a close relationship between *IC* and subjective pleasure by analyzing the data while accounting for a time lag of a few seconds.

This study has some limitations. First, because we analyzed brain activity associated with subjective pleasure and *IC* from the fitting values of each musical piece for each participant, we could not observe changes in brain activity over time for subjective pleasure and *IC*. While different oscillatory activities and brain regions are involved in the anticipation and consumption phases of the reward processing mechanism, our results suggest an association with the consumption phase of reward processing. However, a better understanding of reward processing mechanisms may be achieved by analyzing the time-varying relationships among subjective pleasure, *IC*, and EEG power values across different frequency bands for each musical piece. Reward processing mechanisms have been investigated in many studies using oscillatory activity in terms of monetary rewards; however, comparatively less research has been conducted on musical rewards. Therefore, further studies in this area are required. Second, the oscillatory activity within each frequency band associated with musical rewards and prediction error (*IC*) has been elucidated; however, the integration of neuroimaging techniques is required to identify the associated brain regions. This integration will provide greater clarity on the brain regions involved, the timing of their involvement, and how these regions function in the mechanisms of musical reward processing. Third, musical pleasure has been associated with the *IC* for melody and harmony; however, acoustic features (such as sensory dissonance, spectral centroids, and spectral complexity) have also been suggested to be involved [16]. Therefore, using the MIRtoolbox of MATLAB to calculate these acoustic features and analyze them with *IC* (for melody/harmony) as predictors of musical pleasure, the extent to which *IC* and each acoustic feature contribute to musical pleasure may be determined. Finally, as the sample size in this study was small, future studies could improve the generalizability of the findings by recruiting a larger number of participants who meet the criteria through pre-screening with the BMRQ. Future studies on the aforementioned methods will possibly elucidate the neural mechanisms underlying musical rewards induced by prediction errors and the musical features that influence them.

## 5. Conclusions

In this study, we clarified the neural mechanisms underlying musical pleasure induced by prediction errors through oscillatory activity in the brain. The results suggested that an increase in β and γ power in the frontal regions is associated with subjective pleasure and *IC* (for melody/harmony). This finding is consistent with the neural mechanisms of oscillatory activity observed during monetary reward processing. In the case of musical reward processing, as individuals listen to music in real time, when they predict how upcoming acoustic features will change and encounter prediction errors (surprises), it is probable that neural mechanisms involved in attention, reward, and memory are engaged, reflected in β-γ oscillatory activity when musical pleasure is experienced. Therefore, it is suggested that musical pleasure and *IC* (for melody/harmony) are associated and that β and γ oscillatory activities are deeply involved in the neural mechanisms underlying musical pleasure induced by prediction errors.

## Figures and Tables

**Table 1:** Music lists.

Clip	Title	Genre	Category	Length	Vocal or Instrumental	RWC Music ID	Mean Value of IC for Melody	Mean Value of IC for Harmony	Mean Value of Subjective Pleasure
1	In Your Arms	Pop	Pop	3:49	Vocal	RWC-MDB-2001 No.3	3.65	10.2	1.97
2	Hold On	Pop	Ballad	5:15	Vocal	RWC-MDB-2001 No.4	6.39	10.4	1.86
3	Waiting for Your Love	Rock	Rock	3:30	Vocal	RWC-MDB-2001 No.9	5.3	10.5	1.75
4	Suddenly	Dance	Soul/R&B	3:34	Vocal	RWC-MDB-2001 No.27	5.71	11.3	1.52
5	Wind Up	Jazz	Big band	3:24	Instrumental	RWC-MDB-2001 No.28	6.34	11	1.76
6	Dance to the Samba	Latin	Samba	4:11	Vocal	RWC-MDB-2001 No.41	5.68	11.6	1.72
7	Kittenish Tango	Latin	Tango	3:10	Instrumental	RWC-MDB-2001 No.47	4.36	11	1.94
8	Dear John’s Letter	World	Blues	3:57	Vocal	RWC-MDB-2001 No.66	6.94	10.5	1.52

## Data Availability

The raw data supporting the conclusions of this article will be made available by the authors, without undue reservation.
